# Supplementary material for: Temporal multiplexing of perception and memory codes in IT cortex
Source: Nature. 2024 May 15;629(8013):861–8. doi: 10.1038/s41586-024-07349-5 (PMC11111405; doi:10.1038/s41586-024-07349-5)
Supplement: Supplementary file 1 — This file contains Supplementary methods. [file 41586_2024_7349_MOESM1_ESM.pdf]

nature portfolio

https://doi.org/10.1038/s41586-024-07349-5

Supplementary information

Temporal multiplexing of perception and memory codes in IT cortex

In the format provided by the authors and unedited

Methods

Seven male rhesus macaques (Macaca mulatta) of 5-13 years old were used in this study. All procedures conformed to local and US National Institutes of Health guidelines, including the US National Institutes of Health Guide for Care and Use of Laboratory Animals. All experiments were performed with the approval of the Caltech and UC Berkeley Institutional Animal Care and Use Committee.

Visual stimuli

Face patch localizer. The fMRI localizer stimuli contained 5 types of blocks, consisting of images of faces, hands, technological objects, vegetables/fruits, and bodies. Face blocks were presented in alternation with non-face blocks. Each block lasted 24 s blocks (each image lasted 500 ms). In each run, the face block was repeated four times and each of the non-face blocks was shown once. A block of grid-scrambled noise patterns was presented between each stimulus block and at the beginning and end of each run. Each scan lasted 408 seconds. Additional details can be found in[48].

Monkey face model. To generate a large number of monkey faces, we built an active appearance model for monkey faces[49], similar to the method used for human faces in[50]. Images of frontal views of 165 monkey faces were obtained from the following sources: a private database kindly provided by Dr. Katalin Gothard (101 images), the PrimFace database (visiome.neuroinf.jp/primface)[51] (22 images), YouTube videos of macaques (https://www.youtube.com/@ArrozMarisco360) (16 images), documentary movie (Love Is in the Wild Part 3 - A Monkeys Life, https://kwanza.fr/catalogue/love-is-in-the-wild) (3 images), and face images of macaques from our lab (23 images). The "shape" parameters were obtained by manually labelling 59 landmarks on each of the frontal face images. A 2D triangulated mesh was defined on these landmarks. The coordinates of the landmarks of each image were normalized by subtracting the mean and scaling to the same width, and a landmark template was obtained by averaging corresponding landmarks across faces. The "appearance" parameters were obtained by warping each face to the landmark template through affine transform of the mesh. To reduce the dimensionality of the model, principal component analysis was performed on both the coordinates of the landmarks (shape) and pixels of the warped images (appearance) independently. The first 20 PCs of shape and first 100 PCs of appearance were kept for the final model, capturing 96.1% variance in the shape distribution and 98.4% variance in the appearance distribution. We used this model not only to generate unfamiliar monkey faces, but also to compute shape-appearance features of familiar monkey faces (note: these familiar faces were included in the 165-face database). For the latter, we projected the 59 landmarks and projected these onto the shape PCs; we then morphed the landmarks to the standard landmark template and projected the resulting pixels of the warped images onto the 100 appearance PCs.

Stimuli for electrophysiology experiments. Eight different sources of images were used to generate three different stimulus sets (Extended Data Fig. 2).
1) Personally familiar human faces: Frontal views of faces of 9 people in the lab/animal facility who interacted with the subject monkeys on a daily basis.

2) Personally familiar monkey faces: Frontal views of faces of 9 monkeys in our animal facility that were current or previous roommates or cagemates of the subject monkeys, reconstructed using the monkey face model.
3) Personally familiar objects: Images of 8 toys the subject monkeys interacted with extensively.
4) Pictorially familiar monkey faces: Frontal views of faces of 8 monkeys from the PrimFace database (visiome.neuroinf.jp/primface)[51], reconstructed using the monkey face model.
5) Cinematically familiar monkey faces: Frontal views of faces of 19 monkeys from 7 movies clipped from 7 videos from YouTube (https://www.youtube.com/@ArrozMarisco360) and documentary movie (https://kwanza.fr/catalogue/love-is-in-the-wild), reconstructed using the monkey face model.
6) Unfamiliar human faces: 1840 frontal view of faces from various face databases: FERET[52,53], CVL[54], MR2[55], Chicago[56], CelebA[57], FEI (fei.edu.br/~cet/facedatabase.html), PICS (pics.stir.ac.uk), Caltech faces 1999, Essex (Face Recognition Data, University of Essex, UK; http://cswww.essex.ac.uk/mv/allfaces/faces95.html), and MUCT (www.milbo.org/muct). The background was removed, and all images were aligned, scaled, and cropped so that the two eyes were horizontally located at 45% height of the image and the width of the two eyes equaled 30% of the image width using an open-source face aligner (github.com/jrosebr1/imutils).
7) Unfamiliar monkey faces: 1840 images were generated using the monkey face model described above by randomly drawing from independent Gaussian distributions for shape and appearance parameters, following the same standard deviation as real monkey faces for each parameter. Faces with any parameter larger than 0.8 * maximum value found in a real monkey face were excluded to avoid unrealistic faces.
8) Unfamiliar objects: Images of objects were randomly picked from a subset of categories in the COCO dataset (arXiv:1405.0312). The choice of categories was based on two criteria: 1) only categories that our macaque subjects had no experience with (e.g., vehicles) were included, 2) categories with highly similar objects were excluded (e.g., stop signs). The included super-categories were: 'accessory', 'appliance', 'electronic', 'food', 'furniture', 'indoor', 'outdoor', 'sports', and 'vehicle'. 1500 images of objects with area larger than 200^2 pixels were isolated, centered, and scaled to the same width or height, whichever was larger.

We emphasize that due to the difficulty of obtaining a large set of high-quality monkey face images, we used the monkey face model described above to synthesize unfamiliar monkey faces; for consistency, all familiar monkey faces used in this study were also reconstructed using the monkey face model. Thus any differences in responses to familiar versus unfamiliar faces cannot be attributed to use of synthetic stimuli. Values of each feature dimension were normalized by the standard deviation of the feature dimension for analysis purposes.

From these 8 stimulus sources, 3 different stimulus sets were generated:
1) Screening set consisting of 8 or 9 images from 9 different categories (human faces, monkey faces, and objects, each personally familiar, pictorially familiar, or unfamiliar)

(Extended Data Fig. 2a). There were 74 screening stimuli in all; responses to 24 pictorially familiar faces and objects were not shown in Figure 1. For unfamiliar stimuli, 8 novel images were used for each cell or simultaneously recorded group of cells. Each image was presented in random order, centered at the fixation spot, for 150 ms on 150 ms off (gray screen), repeated 5-10 times. The size of each image was 7.2° x 7.2°. Data using the screening stimulus set are shown in Fig. 1, 4b, c, e, f, Extended Data Fig. 3c, 4, 7, 9d, e, 10a-c.

2) Thousand monkey face set, consisting of 1000 unfamiliar (examples shown in Extended Data Fig. 2b) and 36 familiar faces (personally familiar, pictorially familiar, and cinematically familiar faces, Extended Data Fig. 2a, c), presented using the same parameters as the screening set, except for number of repetitions (3-5 times). In addition, the 8 novel unfamiliar faces shown in the screening set were shown again. Data using the stimulus set are shown in Fig. 2, 3, 4d, g, Extended Data Fig. 3e-h, 5 (except PR of E, and TP), 9, 10d-k.

3) Thousand monkey face set 2, to match both low-level and high-level features for familiar vs. unfamiliar faces. It consisted of 36 familiar faces (personally familiar, pictorially familiar, and cinematically familiar faces, Extended Data Fig. 2a, c), 1044 unfamiliar faces (each set of 36 unfamiliar faces were generated by random permutation of shape appearance features of the 36 familiar faces; the distributions of pairwise distance of low-level features of AlexNet layer 1 for familiar vs. unfamiliar faces were checked by Kolmogorov–Smirnov test, 29 sets of unfamiliar faces that were not significantly different in either distribution were used). These stimuli were presented using the same parameters as the screening set, except the number of repetitions was 3-5 times for 1008 unfamiliar faces, and to control for response reliability differences due to familiarity, a higher number of repeats was used for one set of 36 unfamiliar faces as well as for familiar faces (15-25 times). Data from PR of monkey E and TP of monkeys A and E were collected using this stimulus set.

Behavioral task

For electrophysiology and behavior experiments, monkeys were head fixed and passively viewed a screen in a dark room. Stimuli were presented on an LCD monitor (Acer GD235HZ). Screen size covered 26.0° x 43.9°. Gaze position was monitored using an infrared camera eye tracking system (ISCAN) sampled at 120 Hz.

Passive fixation task. All monkeys performed this task for both fMRI scanning and electrophysiological recording. Juice reward was delivered every 2-4 s in exchange for monkeys maintaining fixation on a small spot (0.2° diameter).

Preferential viewing task. Two monkeys were trained to perform this task. In each trial a pair of face images (7.2° x 7.2°) were presented on the screen side by side with 14.4° center distance (Fig. 1b, Extended Data Fig. 3d). Juice reward was given every 2-4 s in exchange for monkeys viewing either one of the images. Each pair of images lasted 10 s. Face pairs were presented in random order. To avoid side bias, each pair was presented twice with side swapped.

Face identification task. A delayed match-to-sample task was used to test performance on face identification in two monkeys. The task was performed using a touch screen in a cage without head fixation. The subject touched a dot at the center of the screen to initiate a trial. In each trial, a face image (the sample) was shown for 1000 ms, followed by a pair of images (the target and the distractor). The subject had 9 seconds to touch the face that matched the sample. A juice reward was given for correct response. The sample was presented at four different blur levels (clear, Gaussian blur standard deviation 5, 10, and 20 pixels), while the target and distractor were presented as clear versions. The target was identical to the sample except for the difference in blur. The task included 30 pairs of familiar-unfamiliar faces and 30 pairs of unfamiliar-unfamiliar faces. For each face pair, the distractor was matched to the target in low-level features (mean luminance, mean contrast, hue distribution, and shape of the face outline). The Euclidean distance in shape appearance feature space between the target and distractor face was the same across familiar and unfamiliar face targets.

MRI scanning and analysis

Subjects were scanned in a 3T TIM (Siemens, Munich, Germany) magnet equipped with AC88 gradient insert. 1) Anatomical scans were performed using a single loop coil at isotropic 0.5 mm resolution. 2) Functional scans were performed using a custom eight-channel coil (MGH) at isotropic 1 mm resolution, while subjects performed a passive fixation task. Contrast agent (Molday ION) was injected to improve signal/noise ratio. Further details about the scanning protocol can be found in[58].

MRI Data Analysis. Analysis of functional volumes was performed using the FreeSurfer Functional Analysis Stream[59] and FSL[60]. Volumes were corrected for motion and undistorted based on acquired field map. Runs in which the norm of the residuals of a quadratic fit of displacement during the run exceeded 5 mm and the maximum displacement exceeded 0.55 mm were discarded. The resulting data were analyzed using a standard general linear model. The face contrast was computed by the average of all face blocks compared to the average of all non-face blocks.

Single-unit recording

Multiple different types of electrodes were used in this study. Single electrodes (Tungsten, 1 Mohm at 1 kHz, FHC) were used to collect most of the data. A Neuropixel prototype probe (128 channel, HHMI) was used to record ML from subject A. A multi-channel stereotrode (64 channel, Plexon S-probe) was used to record AM during muscimol silencing of PR in subject E. A chronic implanted microwire brush array (64 channel, MicroProbes) (McMahon, et al., 2014) was used to record from face patch AM in subject C. The electrode trajectories that could reach the desired targets were planned using custom software[61], and custom angled grids that guided the electrodes to the target were produced using a 3D printer (3D system). Extracelluar neural signals were amplified and recorded using Plexon. Spikes were sampled at 40 kHz. For single channel recorded data, spike sorting was performed manually by clustering of waveforms above a threshold in PCA space using a custom-made software (Kofiko) in Matlab. Multichannel recorded data was automatically sorted by Kilosort2 (github.com/MouseLand/Kilosort2)[62] and manually refined in Phy (github.com/cortex-lab/phy).

Muscimol experiment

To silence face patch PR, 1 µl (5 mg/ml) muscimol (Sigma) was injected into PR at 0.5 µl/min using G33 needle (Hamilton) connected to a 10 µl micro-syringe controlled by a micro-pump (WPI, UltraMicroPump 3). AM cells were recorded both before and 30 min after injection.

Data analysis

All visually-responsive cells were included for analysis. To determine visual responsiveness, a two-sided T-test was performed comparing activity at [-50 0] ms to that at [50 300] ms after stimulus onset. Cells with *p*-value < 0.05 were included.

Face selectivity index

A face selectivity index (FSI) was defined for each cell as:

$$FSI = \frac{r_{face} - r_{non-face}}{r_{face} + r_{non-face}}$$

where r is the average neuronal response in a 50-300 ms window after stimulus onset (Extended Data Fig. 3c).

Population average of response time course

For each cell, responses to the same stimulus category were first averaged in 10 ms time bins, then the responses were baseline-subtracted (using the average response in the time window 0 to 50 ms), and normalized by the maximum response across different stimulus categories after stimulus onset. The normalized responses were finally averaged across cells for each category after smoothing by a Gaussian function with 10 ms standard deviation (Fig. 1g right, Fig. 3b, Extended Data Fig. 4c, 10c right, 10g).

To determine the time point at which responses rose above baseline (e.g., Fig. 1e), we compared the response at each time point to the baseline response (average response over [-50 0] ms) using a one-tailed T-test, and determined the first time point at which $p < 0.01$.

Preferred axis of cells

The preferred axis of cells was computed in two different ways[63]:

Spike-triggered average (STA). The average firing rate of a neuron was computed to each stimulus, either in a full time window [50-300] ms or sliding 50 ms time window after stimulus onset. The STA was defined as:

$$P_{sta} = (\vec{r} - \bar{r})F$$

where $\vec{r}$ is $1 \times n$ vector of the firing rate response to a set of n face stimuli, $\bar{r}$ is the mean firing rate, and F is a $n \times d$ matrix, where each row consists of the d parameters representing each face stimulus in the feature space.

Linear regression/Whitened STA. For a small sample of stimuli, e.g., 36 familiar faces, the features are not necessarily white (i.e., uncorrelated). As a control, to ensure that the difference in STA observed in Fig. 2b, c was not due to mismatched feature distributions between familiar and unfamiliar faces, we repeated our main analysis using a whitened STA (Extended Data Fig. 9a, bottom) as follows:

$$P_{lin} = (\vec{r} - \bar{r})F(F^T F)^{-1}$$

For all figures, we used 20 dimensions to compute the preferred axis (first 10 shape and first 10 appearance dimensions).

Principal orthogonal axis

The principal orthogonal axis was defined as the *longest* axis orthogonal to its preferred axis. First, for each of the 1000 unfamiliar face images represented as d-dimensional vector ($\vec{f_d}$) in face feature space, its component along the preferred axis (P) of the cell was subtracted

$$\vec{f}_{d-1} = \vec{f_d} - (\vec{f_d} \cdot P/|P|^2)P.$$

Then principal component analysis was performed on the set of 1000 vectors ($\vec{f}_{d-1}$), and the principal orthogonal axis was the first principal component.

Quantifying significance of axis tuning

For each cell, we compared the explained variance by the axis model to a distribution of explained variances computed for data in which stimulus identities were shuffled (1000 repeats). We considered axis tuning significant if the frequency of a higher explained variance in the shuffle distribution was less than 5% (Extended Data Fig. 3e, g).

Quantifying consistency of preferred axis

For each cell, the stimuli were randomly split into two halves, and a preferred axis was calculated using responses to each subset. Then, the Pearson correlation (r) was calculated between the two. This process was repeated 100 times, and the consistency of preferred axis for the cell was defined as the average r value across 100 iterations (Extended Data Fig. 3f, h).

Face feature decoding and reconstruction

To decode face features, firing rates after stimulus onset in a chosen time window (see Fig. 2d, e legend) were first averaged across multiple repeats of the same stimulus, then linear regression was performed on a training set of 999 unfamiliar faces to compute the linear mapping from population response vector $\vec{r}$ to face feature vector $\vec{f}$:

$$\vec{f} = M\vec{r}$$

The decoding was performed on the remaining one unfamiliar and all familiar faces using this mapping *M*. Decoding accuracy was measured by (i) the correlation coefficient between decoded and actual face features (Fig. 2d), (ii) the mean square error between decoded and actual face features (Fig. 4g). For both methods, the decoding accuracy for unfamiliar faces was computed 1000 times through leave-one-out cross validation.

To reconstruct faces (Fig. 2e), we built a face feature decoder as above using responses to unfamiliar faces, computed either in a short ([120 170] ms) or long ([220 270] ms) latency window.

Face identity decoding

To decode face identity (Fig.4b), firing rates after stimulus onset in a chosen time window of each trial were randomly split in half and averaged. Then a multi-class linear SVM decoder was trained to classify each face identity for 30 familiar or 30 unfamiliar feature-matched (Extended Data Fig. 8) faces separately, using one half for training and testing on the other half. This was repeated 20 times.

Familiarity decoding

Firing rates after stimulus onset in a chosen time window (stated for each particular case in the figure legends) were first averaged across multiple repeats of the same stimulus, then the decoding accuracy was obtained as the average of leave-one-out cross-validated linear SVM decoding. For the thousand face set, the training sample was balanced by randomly subsampling 36 unfamiliar faces, repeated 10 times (Fig. 3c).

Centroid shift analysis

To determine the time when the shift of the neural representation centroids for familiar and unfamiliar faces provided familiarity discriminability (Fig. 3d), population responses (50 ms sliding time window, step size 10 ms) to all 36 familiar faces and randomly subsampled 36 unfamiliar faces were first projected to the axis connecting neural centroids of familiar and unfamiliar faces. Then *d'* was computed for the projected values:

$$d' = \frac{\mu_{familiar} - \mu_{unfamiliar}}{\sqrt{\frac{1}{2}(\sigma^2_{familiar} + \sigma^2_{unfamiliar})}}$$

Here μ and σ are the mean and variance of the projected values, respectively. The computation was repeated 10 times for each random subsampling of 36 unfamiliar faces. Chance level *d*' was estimated by randomly shuffling the population responses to each face 10 times. The time when d' was significantly higher than chance was determined by one-tailed T-test ($p < 0.01$, $n = 10$).

Analysis of orthogonality between familiarity and face feature decoding axes

To determine the cosine similarity between familiarity and face feature decoding axes (Fig. 3f), we obtained the familiarity decoding axis as described above in the section on "Familiarity decoding." For unfamiliar faces, we obtained the face feature decoding axis as described in the section above on "Face feature decoding and reconstruction." For familiar faces, we obtained the face feature decoding axis by computing the pseudo-inverse of the face feature encoding axis (necessary due to the small number of familiar faces); we obtained the latter as described above in the section on "Preferred axis of cells" (using the STA).

Computing normalized firing rate changes

Normalized firing rate change in Fig. 4e was computed as follow:

$$\frac{R_{after} - R_{before}}{R_{after} + R_{before}}$$

where R_{before} is the mean firing rate within 50-300 ms after stimulus onset before Muscimol injection, and R_{after} is the same for after muscimol injection.

Matching face feature distributions

We wanted to ensure that the difference in preferred axis (Fig. 2b, c) and the difference in pairwise distance in the neural state space (Extended Data Fig. 10j, k) were not due to mismatched feature distributions between familiar and unfamiliar faces. To this end, we identified a *feature-matched subset* of 30 familiar and 30 unfamiliar faces. For the top 20 face features, these two face sets were matched in feature variance (Extended Data Fig. 8a), distribution of pairwise face distances in feature space (Extended Data Fig. 8b), and distribution of each feature (Extended Data Fig. 8c). This was achieved by searching for a subset of faces that minimized the following cost function:

$$C = C_{var} + C_p$$

The first term evaluated the difference of variance:

$$C_{var} = \sum_{i=1}^{n} (v_{familiar}(i) - v_{unfamiliar}(i))^2/n + \left| \sum_{i=1}^{n} v_{familiar}(i)/n - \sum_{i=1}^{n} v_{unfamiliar}(i)/n \right|$$

where *v(i)* is the variance of the *ith* feature, *n* = 20 is the number of features. It is the sum of mean square error and absolute value of mean difference between the variance of each feature.

The second term ensured the distributions in consideration are not significantly different, which was measured by the *p* values of K-S test being larger than 0.05:

$$C_p = g(min(p_D, p_1, \dots, p_n))$$

$$g(x) = \begin{cases} 1/(x + 0.001) & x < 0.05 \\ 0 & x \geq 0.05 \end{cases}$$

where p_D is the *p* value of K-S test between distributions of pairwise face distances for familiar vs. unfamiliar, p_i is the *p* value of K-S test between distributions of the *ith* feature for familiar vs. unfamiliar.

The optimization was performed using a gradient-descent-like algorithm: in each iteration dC was estimated by removing or adding each face, and the change that decreased C the most was applied, until C did not decrease anymore. To balance the number of familiar and unfamiliar faces in the result, we set a minimum number of familiar faces (23-36). When the number was chosen to be 30, the resulting number of unfamiliar faces also happened to be 30.

Finally, we confirmed that for the resulting set of 30 familiar and 30 unfamiliar faces, the faces were indeed feature matched (Extended Data Fig. 8), and the axis model explained similar amounts of variance for both familiar and unfamiliar face responses.

In Extended Data Fig. 2b, to demonstrate the diversity of faces in the 1000 face set and the capability to match each of our familiar face sets, we used the same matching method with different subsets of familiar faces.

Face feature sensitivity analysis

To test whether tuning to familiar faces can be explained by loss of tuning to certain feature dimensions, we computed the sensitivity to each feature for 36 familiar faces or a random subsample of 36 unfamiliar faces. Sensitivity was measured as the slope of the linear regression of feature value vs. neuronal firing rate.

Test simple nonlinear model

To test whether a logistic output nonlinearity can explain apparent axis change, we compared the explained variance of an axis model followed by a linear function or logistic function. The 36 unfamiliar or 36 familiar faces were first projected to a preferred axis computed from responses to 964 unfamiliar faces, then the projected value vs. neural responses were fitted by either a linear function or a logistic function ($f(x) = \frac{L}{1+e^{-k(x-x_0)}}$).

Additional details for figures

Figure 1b. Data combined from two test sessions on two different days for each subject. 36 familiar-unfamiliar faces pairs (18 unfamiliar faces sampled from 1000 unfamiliar faces, and the other 18 unfamiliar faces were novel faces never seen before).
Figure 1c. Firing rate responses were normalized by dividing the maximum response across the stimuli for each cell.
Figure 2a. The 8 random unfamiliar faces indicated by the green dots were excluded from calculation of the preferred axis of the cells here.
Figure 2b. In computing the cosine similarity of the unfamiliar vs. unfamiliar condition for stimulus set #2 (most AM and PR) random sample of subsets of 36 unfamiliar faces (100 repeats) were pooled together. For stimulus set #3 (PR of monkey E and TP), to control for response reliability difference of familiarity, the set of 36 unfamiliar faces that presented at same high repeats as familiar faces were used. Same for Extended Data Fig. 5a, b.
Figure 2c. Computed using a 50 ms sliding time window, step size 10 ms.
Figure 2d. The decoder was trained on large set of unfamiliar faces, the performance was measured by the correlation coefficient between actual and decoded face feature vectors, computed using a 50 ms sliding time window, step size 10 ms.
Figure 3a. Responses normalized same as Figure 1c. Number of cells, AM n = 134, PR n = 76, TP n = 197.
Figure 3b. Significance criteria, $p < 0.001$.
Figure 3c. Accuracy computed using a 50 ms sliding time window, step size 10 ms. Chance level was obtained using shuffled data. Significant criteria, $p < 0.01$.
Figure 3d. Distance computed using a 50 ms sliding time window, step size 10 ms. Significant criteria, $p < 0.01$. For stimulus set #2 (most AM and PR) random sample of subsets of 36 unfamiliar faces (10 repeats) were used. For stimulus set #5 (PR of monkey E and TP), to control for response reliability difference of familiarity, the set of 36 unfamiliar faces that presented at same high repeats as familiar faces were used.
Figure 3e. Significant criteria, $p < 0.05$.
Figure 4f, g. Response time window 50-300 ms.

Statistics and Reproducibility

Experiment 1 (screening stimuli) was repeated in two different animals for AM, TP, and ML, in three different animals for PR (Extended Data Fig. 4a-c).
Experiment 2 (thousand monkey faces) was repeated in three different animals for AM and PR, in two different animals for TP and ML (Extended Data Fig. 5a-c).
Muscimol silencing experiments were repeated in two different animals (A, E) with similar results. Fig. 4 showed results pooled from the two animals.
View preference test were repeated in two different animals (Fig. 1b, Extended Data Fig. 3d), face identification task were repeated in two different animals (Extended Data Fig. 3b).
Additional information for statistical test can be found in Supplementary Table 1.

References

48 Tsao, D. Y., Freiwald, W. A., Knutsen, T. A., Mandeville, J. B. & Tootell, R. B. Faces and objects in macaque cerebral cortex. *Nature neuroscience* **6**, 989 (2003).

49 Cootes, T. F., G.J., E. & Taylor, C. J. Active appearance models. *IEEE Transactions on pattern analysis and machine intelligence* **23**, 681-685 (2001).

50 Chang, L. & Tsao, D. Y. The code for facial identity in the primate brain. *Cell* **169**, 1013-1028. e1014 (2017).

51 The face images used in this study are provided by PrimFace database: http://visiome.neuroinf.jp/primface, funded by Grantin-Aid for Scientific research on Innovative Areas, "Face Perception and Recognition" from Ministry of Education, Culture, Sports, Science, and Technology (MEXT), Japan.

52 Phillips, P. J., Wechsler, H., Huang, J. & Rauss, P. J. The FERET database and evaluation procedure for face-recognition algorithms. *Image Vision Comput* **16** 295-306 (1998).

53 Phillips, P. J., Moon, H., Rizvi, S. A. & Rauss, P. J. The FERET evaluation methodology for face-recognition algorithms. *Ieee T Pattern Anal* **22,** , 1090-1104 (2000).

54 Solina, F., Peer, P., Batagelj, B., Juvan, S. & Kovac, J. in *Conference on Computer Vision / Computer Graphics Collaboration for Model-based Imaging, Rendering, image Analysis and Graphical special Effects* Vol. 10 (2003).

55 Strohminger, N. *et al.* The MR2: A multi-racial, mega-resolution database of facial stimuli. *Behav Res Methods* **48**, 1197-1204 (2016).

56 Ma, D. S., Correll, J. & Wittenbrink, B. The Chicago face database: A free stimulus set of faces and norming data. *Behav Res Methods* **47**, 1122-1135 (2015).

57 Yang, S., Luo, P., Loy, C. C. & Tang, X. in *IEEE International Conference on Computer Vision 9.*

58 Ohayon, S., Freiwald, W. A. & Tsao, D. Y. What makes a cell face selective? The importance of contrast. *Neuron* **74**, 567-581 (2012).

59 Reuter, M. & Fischl, B. Avoiding asymmetry-induced bias in longitudinal image processing. *Neuroimage* **57**, 19-21 (2011).

60 Jenkinson M, B. C., Behrens TE, Woolrich MW, Smith SM. FSL. *Neuroimage* **62**, 782-790, doi:10.1016/j.neuroimage.2011.09.015 (2012).

61 Ohayon, S. & Tsao, D. Y. MR-guided stereotactic navigation. *Journal of neuroscience methods* **204**, 389-397 (2012).

62 Pachitariu, M., Sridhar, S., & Stringer, C. Solving the spike sorting problem with Kilosort. *bioRxiv* **2023-01.** (2023).

63 She, L., Benna, M., Shi, Y., Fusi, S., & Tsao, D. Data and code for "Temporal multiplexing of perception and memory codes in IT cortex. She et al. Nature 2024" [Data set]. *Zenodo*, doi:https://doi.org/10.5281/zenodo.10460607 (2024).
