# Supplementary material for: Temporal multiplexing of perception and memory codes in IT cortex
Source: Nature. 2024 May 15;629(8013):861–8. doi: 10.1038/s41586-024-07349-5 (PMC11111405; doi:10.1038/s41586-024-07349-5)
Supplement: Supplementary file 2 — Reporting Summary [file 41586_2024_7349_MOESM2_ESM.pdf]

nature portfolio

Corresponding author(s): Doris Tsao

Last updated by author(s): 4/20/2024

Reporting Summary

Nature Portfolio wishes to improve the reproducibility of the work that we publish. This form provides structure for consistency and transparency in reporting. For further information on Nature Portfolio policies, see our Editorial Policies and the Editorial Policy Checklist.

Statistics

For all statistical analyses, confirm that the following items are present in the figure legend, table legend, main text, or Methods section.

n/a	Confirmed	
☐	☒	The exact sample size (n) for each experimental group/condition, given as a discrete number and unit of measurement
☐	☒	A statement on whether measurements were taken from distinct samples or whether the same sample was measured repeatedly
☐	☒	The statistical test(s) used AND whether they are one- or two-sided<br>*Only common tests should be described solely by name; describe more complex techniques in the Methods section.*
☒	☐	A description of all covariates tested
☒	☐	A description of any assumptions or corrections, such as tests of normality and adjustment for multiple comparisons
☐	☒	A full description of the statistical parameters including central tendency (e.g. means) or other basic estimates (e.g. regression coefficient) AND variation (e.g. standard deviation) or associated estimates of uncertainty (e.g. confidence intervals)
☐	☒	For null hypothesis testing, the test statistic (e.g. F, t, r) with confidence intervals, effect sizes, degrees of freedom and P value noted<br>*Give P values as exact values whenever suitable.*
☒	☐	For Bayesian analysis, information on the choice of priors and Markov chain Monte Carlo settings
☒	☐	For hierarchical and complex designs, identification of the appropriate level for tests and full reporting of outcomes
☐	☒	Estimates of effect sizes (e.g. Cohen's d, Pearson's r), indicating how they were calculated

Our web collection on statistics for biologists contains articles on many of the points above.

Software and code

Policy information about availability of computer code

Data collection	Electrophysiological signals were collected by Plexon system. Gaze position was monitored using ISCAN system. All other experimental parameters were recorded using a custom software Kofiko (https://github.com/shayo/Kofiko) (version: Feb/4/2014). The electrode trajectories were planned using custom software (https://github.com/shayo/Planner) (Revision 93 Feb/19/2014).
Data analysis	Functional imaging data are prcoessed with Freesurfer and FSL. Multichannel recorded electrophysiological data was automatically sorted by Kilosort2 (github.com/MouseLand/Kilosort2) and manually refined in Phy (github.com/cortex-lab/phy). Custom code written in MATLAB was used for analysis. The code that reproduce the core results are available at https://doi.org/10.5281/zenodo.10460607. All other code is available from the lead corresponding author upon reasonable request.

For manuscripts utilizing custom algorithms or software that are central to the research but not yet described in published literature, software must be made available to editors and reviewers. We strongly encourage code deposition in a community repository (e.g. GitHub). See the Nature Portfolio guidelines for submitting code & software for further information.

Data

Policy information about availability of data

All manuscripts must include a data availability statement. This statement should provide the following information, where applicable:

- Accession codes, unique identifiers, or web links for publicly available datasets
- A description of any restrictions on data availability
- For clinical datasets or third party data, please ensure that the statement adheres to our policy

The data set of neural responses to thousand monkey face set are available at https://doi.org/10.5281/zenodo.10460607.
PrimFace database: http://visiome.neuroinf.jp/primface
FERET database: https://www.nist.gov/itl/products-and-services/color-feret-database
CVL Face Database: http://www.lrv.fri.uni-lj.si/facedb.html
MR2 face database: https://osf.io/skbq2/
Chicago Face Database: https://www.chicagofaces.org/
CelebA CelebFaces Attributes Dataset: https://mmlab.ie.cuhk.edu.hk/projects/CelebA.html
FEI Face Database: fei.edu.br/~cet/facedatabase.html
PICS Psychological Image Collection at Stirling: pics.stir.ac.uk
Caltech faces 1999: https://data.caltech.edu/records/6rjah-hdv18
Essex Face Recognition Data: http://cswww.essex.ac.uk/mv/allfaces/faces95.html
The MUCT Face Database: www.milbo.org/muct
All other data are available from the lead corresponding author upon reasonable request.

Research involving human participants, their data, or biological material

Policy information about studies with human participants or human data. See also policy information about sex, gender (identity/presentation), and sexual orientation and race, ethnicity and racism.

Reporting on sex and gender	N/A
Reporting on race, ethnicity, or other socially relevant groupings	N/A
Population characteristics	N/A
Recruitment	N/A
Ethics oversight	N/A

Note that full information on the approval of the study protocol must also be provided in the manuscript.

Field-specific reporting

Please select the one below that is the best fit for your research. If you are not sure, read the appropriate sections before making your selection.

☒ Life sciences ☐ Behavioural & social sciences ☐ Ecological, evolutionary & environmental sciences

For a reference copy of the document with all sections, see nature.com/documents/nr-reporting-summary-flat.pdf

Life sciences study design

All studies must disclose on these points even when the disclosure is negative.

Sample size	Sample sizes were chosen in a manner commensurate with similar previous studies.
Data exclusions	We recorded from every neuron encountered. Only visual responsive units were considered for further analysis.
Replication	Results were replicated across 2-3 different animals for each experiment independently.
Randomization	The visual stimuli were shown in a random order. Organisms random allocation is not relevant to this study, different subjects were used to repeat the same experimental condition.
Blinding	Investigators were not blinded to experimental groups due to the nature of the experiments.

Reporting for specific materials, systems and methods

We require information from authors about some types of materials, experimental systems and methods used in many studies. Here, indicate whether each material, system or method listed is relevant to your study. If you are not sure if a list item applies to your research, read the appropriate section before selecting a response.

Materials & experimental systems

n/a	Involved in the study	
☒	☐	Antibodies
☒	☐	Eukaryotic cell lines
☒	☐	Palaeontology and archaeology
☐	☒	Animals and other organisms
☒	☐	Clinical data
☒	☐	Dual use research of concern
☒	☐	Plants

Methods

n/a	Involved in the study	
☒	☐	ChIP-seq
☒	☐	Flow cytometry
☐	☒	MRI-based neuroimaging

Animals and other research organisms

Policy information about studies involving animals; ARRIVE guidelines recommended for reporting animal research, and Sex and Gender in Research

Laboratory animals	Seven male rhesus macaques (Macaca mulatta) of 5-13 years old were used in this study.
Wild animals	The study did not involve wild animals.
Reporting on sex	This study was conducted using only male animals.
Field-collected samples	The study did not involve field-collected samples.
Ethics oversight	All procedures conformed to local and US National Institutes of Health guidelines, including the US National Institutes of Health Guide for Care and Use of Laboratory Animals. All experiments were performed with the approval of the Caltech and UC Berkeley Institutional Animal Care and Use Committee.

Note that full information on the approval of the study protocol must also be provided in the manuscript.

Magnetic resonance imaging

Experimental design

Design type	Block design
Design specifications	Each block lasted 24 s blocks (each image lasted 500 ms). In each run, the face block was repeated four times and each of the non-face blocks was shown once. A block of grid-scrambled noise patterns was presented between each stimulus block and at the beginning and end of each run. Each scan lasted 408 seconds.
Behavioral performance measures	Subjects' eye position was monitored using an infrared eye tracking system (ISCAN). Juice reward was delivered every 2-4 s in exchange for maintaining fixation on a small spot (0.2 degree)

Acquisition

Imaging type(s)	Functional and anatomical imaging
Field strength	3 Tesla
Sequence & imaging parameters	T1-weighted anatomical volumes were measured with MP-RAGE sequence(TR 2,300 ms; IR 1,100 ms; TE 3.37 ms; 0.5 mm isotropic voxels) . EPI volumes were acquired in an AC88 gradient insert (Siemens) TR was 2000 ms,TE was 17 ms, voxels were 1 × 1 × 1 mm with an no gap between slices. Matrix size was (96, 96, 64) (read [x], phase [y], slice [z]), the field of view was 96 × 96 mm in-plane. Flip angle was 80°
Area of acquisition	Whole brain

Diffusion MRI ☐ Used ☒ Not used

Preprocessing

Preprocessing software	Analysis of functional volumes was performed using the FreeSurfer Functional Analysis Stream (Massachusetts General Hospital). Volumes were corrected for motion and undistorted based on acquired field map.
Normalization	No normalization needed as analysis only compare data from the same scan.

Normalization template	We did not normalize any imaging data into template. All the analysis were done in the single subject's original space.
Noise and artifact removal	We remove the linear or quadratic trends in the timeseries.
Volume censoring	Motion noises were removed by putting the motion parameters as the regressors in the GLM analysis.

Statistical modeling & inference

Model type and settings	The analysis used only first-level analysis.
Effect(s) tested	We ran t-tests between different conditions within each single subject.

Specify type of analysis: ☒ Whole brain ☐ ROI-based ☐ Both

Statistic type for inference (See Eklund et al. 2016)	All the analyses were done using voxel-wise inference.
Correction	We did not apply any multiple-comparison correction in the fMRI imaging analysis.

Models & analysis

n/a	Involved in the study
☒	☐ Functional and/or effective connectivity
☒	☐ Graph analysis
☒	☐ Multivariate modeling or predictive analysis
